# A new mass spectrometry-based method for the quantification of histones in plasma from septic shock patients

**DOI:** 10.1038/s41598-017-10830-z

**Published:** 2017-09-06

**Authors:** J. L. García-Giménez, C. Romá-Mateo, N. Carbonell, L. Palacios, L. Peiró-Chova, E. García-López, M. García-Simón, R. Lahuerta, C. Gimenez-Garzó, E. Berenguer-Pascual, M. I. Mora, M. L. Valero, A. Alpízar, F. J. Corrales, J. Blanquer, F. V. Pallardó

**Affiliations:** 1 Center for Biomedical Network Research on Rare Diseases (CIBERER), Institute of Health Carlos III, Valencia, Spain; 2 Department of Physiology, Faculty of Medicine and Dentistry, University of Valencia, Valencia, Spain; 3 INCLIVA Biomedical Research Institute, Valencia, Spain; 4 Epigenetics Research Platform, CIBERER/UV, Valencia, Spain; 5 Intensive Care Unit, Clinical University Hospital of Valencia, Valencia, Spain; 6 INCLIVA Biobank, INCLIVA Biomedical Research Institute, Valencia, Spain; 7 Department of Hepatology, Proteomics laboratory, CIMA, University of Navarra; Ciberhed; Idisna; PRB2, ProteoRed-ISCIII, Pamplona, Spain; 8 Central Service for Experimental Research (SCSIE), University of Valencia, Burjassot, Spain; 9 Proteomics Unit, Centro Nacional de Biotecnología (CSIC); PRB2, ProteoRed-ISCIII, Madrid, Spain; 10 Faculty of Biomedical and Health Sciences, Universidad Europea de Valencia, Valencia, Spain

## Abstract

The aim of this study was to develop a novel method to detect circulating histones H3 and H2B in plasma based on multiple reaction monitoring targeted mass spectrometry and a multiple reaction monitoring approach (MRM-MS) for its clinical application in critical bacteriaemic septic shock patients. Plasma samples from 17 septic shock patients with confirmed bacteraemia and 10 healthy controls were analysed by an MRM-MS method, which specifically detects presence of histones H3 and H2B. By an internal standard, it was possible to quantify the concentration of circulating histones in plasma, which were significantly higher in patients, and thus confirmed their potential as biomarkers for diagnosing septic shock. After comparing surviving patients and non-survivors, a correlation was found between higher levels of circulating histones and unfavourable outcome. Indeed, histone H3 proved a more efficient and sensitive biomarker for septic shock prognosis. In conclusion, these findings suggest the accuracy of the MRM-MS technique and stable isotope labelled peptides to detect and quantify circulating plasma histones H2B and H3. This method may be used for early septic shock diagnoses and for the prognosis of fatal outcomes.

## Introduction

Sepsis is defined as a life-threatening organ dysfunction through a dysregulated host response to infection^1^. Septic shock implies a sepsis subset in which particularly profound circulatory, cellular and metabolic abnormalities are associated with a higher risk of mortality. Sepsis is a major healthcare problem that affects millions of people, and its incidence is increasing owing to ageing populations, immuno-senescence and the resulting impaired immunity. It is the most frequent cause of mortality in most intensive care units (ICUs)^2^. Despite its worldwide importance, and it being considered a public health concern that accounts for more than $20 billion (5.2% of total US hospital costs) in 2011^3^, public awareness of sepsis is poor.

Short-term sepsis mortality ranges between 10% and 40%, and even reaches 30–60% in septic shock^4^. The advances made in therapy and improvements to treatments have proved effective in reducing mortality, but short-term mortality is still very high^5^. Hence monitoring sepsis will largely benefit from new affordable biomarkers, as suggested by Pierrakos and Vincent^6^.

Presence of circulating histones in relation to septic processes has been previously studied, and several works have shown a direct correlation between presence of specific histones in plasma and the symptoms that derive from prolonged sepsis duration^7, 8^. Circulating histones are found in the blood of healthy individuals at low concentrations, but their levels rise mainly in patients who suffer from severe trauma^9^, systemic inflammation^10^, sepsis or tissue damage^11^. It is noteworthy that histone proteins seem to have an interesting cytotoxic potential^12–14^, which hence worsens the complications inherent to sepsis evolution^15^. However, no definitive consensus has been reached on the specific concentration levels for each histone type in relation to their pathogenicity. Specific histone levels and their capacity to monitor sepsis progression, as well as the correlation with effectiveness of treatments, have never been fully elucidated.

Immunoassays (IAs) run to detect the free histone proteins that circulate in plasma have been formerly used^16, 17^, but the obtained results show poor reproducibility, wide error ranges and low sensitivity^18^. Semi-quantitative IAs also bear many flaws; e.g., poor concordance between assays, differences in reproducibility and variable cross-reactivity^19^. A variety of antigen-antibody interferences can also occur in IAs, which can generate erroneous results and cause clinical side effects due to erroneous diagnosis and inadequate treatments^20, 21^. Replacing IAs with mass spectrometry-based methods could increase the sensitivity and specificity of biomarkers^22^. In line with this, targeted mass spectrometry (MS) with specific heavily labelled Spike-In peptides is an efficient and sensitive approach for protein identification and quantification^23^.

In the present work, we propose a novel method to detect circulating histones H3 and H2B in plasma from septic shock patients with bacteraemia based on multiple reaction monitoring targeted mass spectrometry (MRM-MS). Recently, Kusebauch *et al*. used this assay to identify and quantify 99.7% of the 20,277 annotated human proteins with high sensitivity and high selectivity by this widely accessible, sensitive and robust targeted mass spectrometry technique: selected reaction monitoring and SIL (spiked-in labelled) proteotypic peptides, which shows the potential of this method^24^.

Our method allows histone quantification using an internal standard and shows strong specificity and sensitivity values.

## Results

### MRM analysis of H2B and H3

A selection of histone peptides was firstly assayed to choose the best candidates for the Spiked-In preparations and posterior MRM-MS measures in blood samples. Peptides LLLPGELAK and STELLIR, which respectively derived from the tryptic digestion of purified H2B and H3, were selected upon experimental observations in LC-MS/MS experiments. This is a critical point to design MRM assays because different peptides from the same protein can offer a vast variety of MS responses, and could produce interferences in plasma samples^25^. The unique peptide sequences finally chosen exhibited good chromatographic parameters, optimal ionisation efficiency and high MS/MS quality, and showed large concentration linearity for the signal (data not shown). Both sequences were used to spike the plasma samples from both the septic shock patients and healthy subjects.

### General description of cohorts

Ten healthy controls were recruited; their median age was 42 years (20–56) and males accounted for 70%. Cases consisted in 17 patients with a clinical diagnosis of bacteriaemic septic shock. The median age was 67 years (37–85) and male patients accounted for 67% of cases. A median APACHE II score for the septic shock patients was 25 (14–44), and the median SOFA score value was 10 (3–19). The detailed clinical data from the septic shock patients, subclassified as survivors and non-survivors, is summarised in Table 1.

**Table 1:** Clinical features of the patients with bacteraemia-septic shock.

Participants’ characteristics	SURVIVORS (n = 9)	NON-SURVIVORS (n = 8)	*p* value
Age (years) (mean ± SD)	66 ± 12	63 ± 14	0.72
Male gender (%)	7 (78)	5 (62)	0.43
APACHE II score (mean ± SD)	**21 ± 4**	**29 ± 10**	**0.05**
Charlson Index > 3 (%)	4 (44)	4 (50)	0.6
SOFA score 1^st day^ (mean ± SD)	9 ± 2	11 ± 5	0.5
Antibiotic 2 weeks prior to admission (%)	2 (22)	1 (12.5)	0.54
Hospital admission in the previous 3 weeks	2 (22)	3 (37)	0.53
Infection source (%)			
Abdominal/urologic	8 (88.8)	4 (50)	
Respiratory	0	2 (25)	
SSTI/bone infection	0	2 (25)	
Unknown	1 (11)	0	0.09
Microorganisms (%)			
MDR^a^	0	2 (25)	
Non-MDR^b^	9 (100)	5 (62.5)	
* Candida spp*.	0	1 (12.5)	0.17
Organ support therapy (%)			
Antimicrobial in first hour	6 (67)	4 (50)	0.48
Corticosteroid therapy	4 (52)	6 (75)	0.42
Crystalloids (mL) (mean ± SD)	1,811 ± 382	1,850 ± 111	0.93
Vasopressor therapy	9 (100)	8 (100)	NS
RRT	**3 (33)**	**7 (87)**	**0.03**
Mechanical ventilation	**2 (22)**	**7 (87)**	**0.01**
Lactate Clearance^c^	**8 (89)**	**3 (37.5)**	**0.04**
ICU LOS (days) (mean ± SD)	7 ± 7	11 ± 11	0.47
Hospital LOS (days) (mean ± SD)	18 ± 15	17 ± 21	0.90
White blood cells (mean ± SD)	19,464 ± 10,731	15,207 ± 12,200	0.45
CRP (mg/l) (mean ± SD)	288 ± 51	279 ± 77	0.90
Procalcitonin (ng/mL) (mean ± SD)	61 ± 15	22 ± 13	0.07
Lactate 1^st hour^ (mmol/l) (mean ± SD)	5 ± 3	8 ± 6	0.10
Lactate 6 ^hours^ (mmol/l) (mean ± SD)^c^	**3 ± 3**	**6.5 ± 4**	**0.05**
Glucose (mg/dl) (mean ± SD)	142 ± 50	210 ± 100	0.11
Creatinine (mg/dl) (mean ± SD)	2.9 ± 1.5	2.5 ± 1.2	0.58
PaO_2_/FiO_2_ ratio (mean ± SD)	**266 ± 103**	**163 ± 69**	**0.03**
Prothrombin time (seconds) (mean ± SD)	**16.6 ± 2.8**	**20.4 ± 4.7**	**0.05**
Platelets count (mean ± SD)	145,111 ± 101,160	243,875 ± 140,026	0.11
Albumin (g/dl) (mean ± SD)	2.8 ± 0.2	2.6 ± 0.5	0.39

Significant differences (*p* value < 0.05) between groups are highlighted in bold type. SD = standard deviation, APACHE = acute physiology and chronic health evaluation, SOFA = sequential organ failure assessment, SSTI = skin and soft tissue infection, MDR = multidrug resistant microorganisms, RRT = renal replacement therapy, ICU = intensive care unit, LOS = length of stay CRP = C-reactive protein, PaO_2_/FiO_2_ ratio = arterial oxygen partial pressure to fractional inspired oxygen.

^a^MDR (extended Spectrum Betalactamase-producing *Escherichia coli* and Methicillin Resistant *Staphylococcus aureus*)

^b^Non-MDR (*Escherichia coli, Klebsiella pneumoniae, Enterococcus faecalis, Enterococcus faecium, Enterobacter cloacae, Streptococcus mitis* and *Streptococcus pneumoniae*).

^c^Lactate clearance over 10% during the first 6 h of haemodynamic resuscitation.

Cases included nine (53%) survivors and eight (47%) non-survivors. Both groups had similar demographic characteristics, comorbidities upon admission according to the Charlson Index, and severity. However, non-survivors had a higher mean APACHE II score (Table 1). No significant differences were noted in both source of infection and the microorganisms identified in blood samples. Initial management was similar in both septic shock patient groups; survivors and non-survivors received intravenous antimicrobial therapy within the first hour of recognising septic shock and fluid resuscitation (initial administration of at least 20 mL/kg of intravenous crystalloids) to improve haemodynamics in the absence of formal contraindication. According to organ support therapy, all the patients needed vasoactive drugs at the beginning (when the septic shock inclusion criteria were considered) and a patient population higher than 50% was treated with corticosteroids, based on a refractory situation, in both groups. In the patients whose lactate levels lowered by more than 10% after fluid resuscitation, mortality was of 27%, with an odds ratio (OR) of ICU mortality of 0.4 (95%CI: 0.16–1.06) for the patients who cleared this metabolite. No difference was observed for median ICU and hospital lengths of stay between both patient groups. Furthermore, non-survivors required mechanical ventilation and renal replacement therapy more than survivors. The patients’ blood test results on the first day of ICU stay showed few differences between groups. Inflammatory parameters (white blood cells count, C-reactive protein (CRP) and procalcitonin (PCT) levels) were comparable. Significant differences were observed in lactate levels 6 h after therapy for the prothrombin time and arterial oxygen partial pressure to fractional inspired oxygen (PaO_2_/FiO_2_) ratio between both groups. Once again this revealed that the worse situation and more advanced organ dysfunction were evident for the non-survivors group.

### Circulating histones H2B and H3 as potential biomarkers for the diagnosis and prognosis of bacteraemia septic shock patients

The levels of histones H2B and H3 were quantified in plasma samples by the aforementioned MRM-MS approach using standard curves with different concentrations of recombinant human histone H2B and H3 and stable isotopically labelled peptides, as described in methods section and in supplementary information. We used stable isotopic Spike-In peptides, which are valid for absolute protein quantification^26^ and MRM-MS-based assays, which allow histone quantification in complex biological matrices^27–29^. Therefore, the use of standard curves, stable isotopically labelled peptides and MRM-MS technique was valid for absolute quantification of circulating histones H2B (supplementary Figure S1) and H3 (supplementary Figure S2).

**Figure 1:**
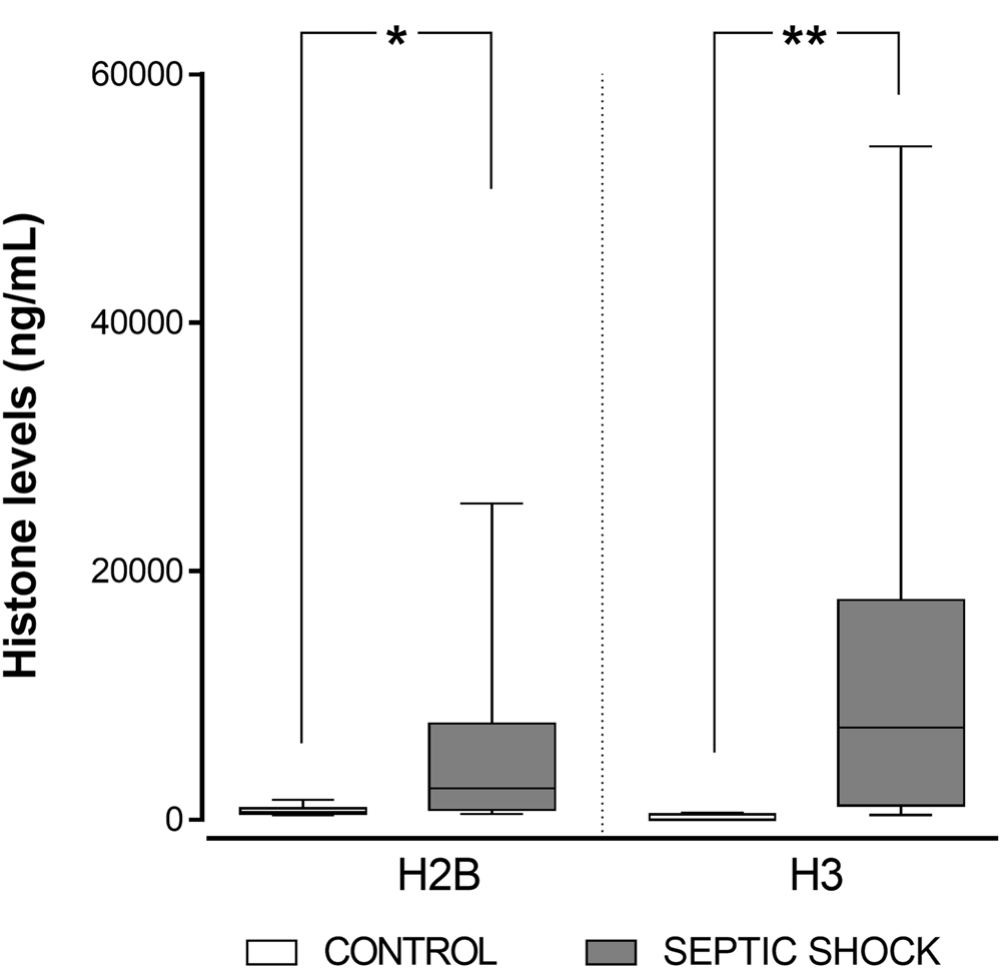
Levels of circulating histones H2B and H3 in plasma from control and septic shock patients. Box and whisker plots represent the H2B and H3 levels in healthy subjects (n = 10) versus the septic shock patients with bacteraemia (n = 17). Boxes denote interquartile ranges, horizontal lines denote medians, and whiskers denote the 10^th^ and 90^th^ percentiles. Levels are expressed as ng/mL of H2B or H3 in plasma. Differences between the two groups were compared using a Student’s *t*-test (*p < 0.05; **p < 0.01).

For histone H2B, the mean level found in the 10 plasma samples of the controls was 720.15 ng/mL (95%CI: 451.23–989.06), which increased up to 5,843.23 ng/mL (95%CI: 1,891.76–9,794.70) in the patients’ 17 plasma samples. The observed differences were statistically significant (p = 0.014), and were even higher for histone H3, where the mean levels were 179.77 ng/mL (95%CI: 7.64–351.89) for the controls and 11,641.33 ng/mL (95%CI: 4,408.04–18,874.62) for the patients (p = 0.004) (Fig. 1).

**Figure 2:**
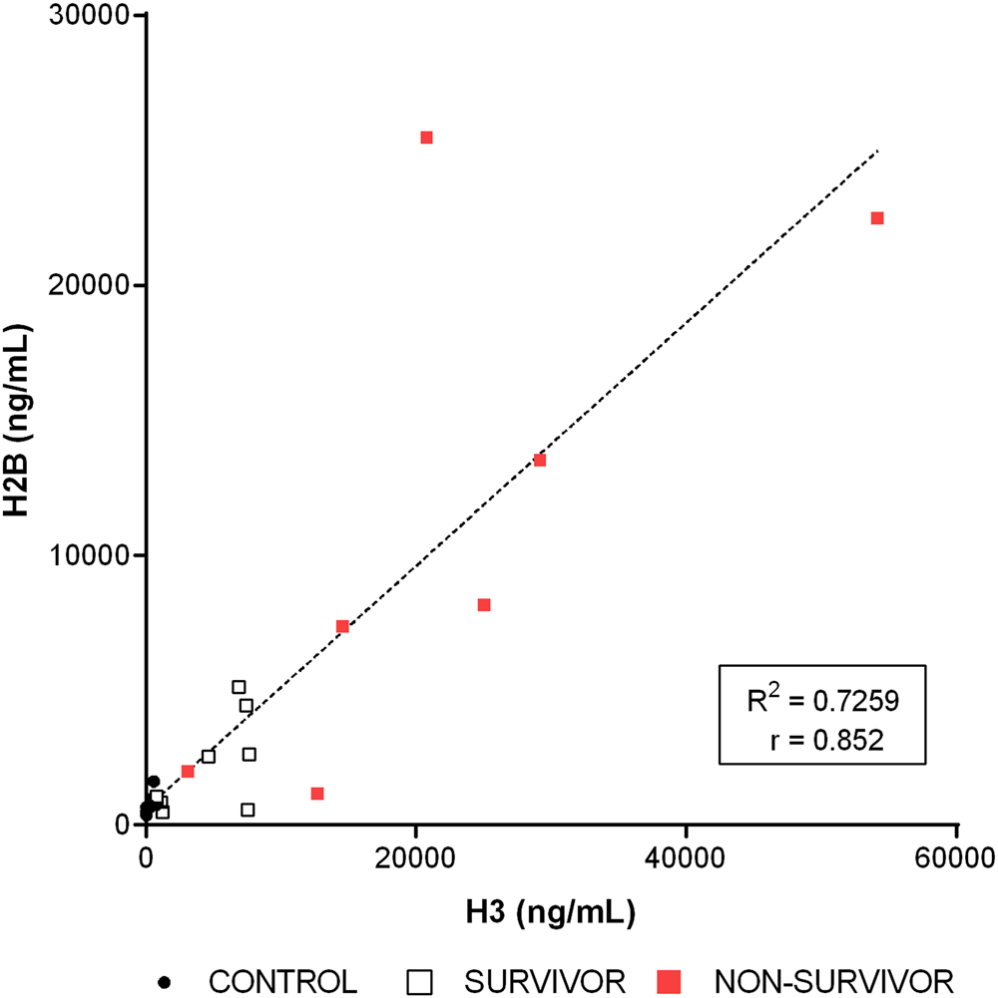
Correlation of the H3 and H2B levels in the plasma samples. Levels of H3 and H2B (ng/mL) measured in the 27 plasma samples taken from the subjects who participated in the study (17 cases, including survivors and non-survivors, 10 controls).

The levels of histones H3 and H2B in plasma correlated positively with each other, as shown in Fig. 2. We also calculated separately correlation between levels of histones in both the control (n = 10) and patient groups (n = 17, including survivors and non-survivors). The results observed were also statistically significant. Pearson´s correlation coefficient for controls was r = 0.856, p < 0.0001; and for patients Pearson´s correlation coefficient was r = 0.824, p < 0.0001) (graphics not shown).

**Figure 3:**
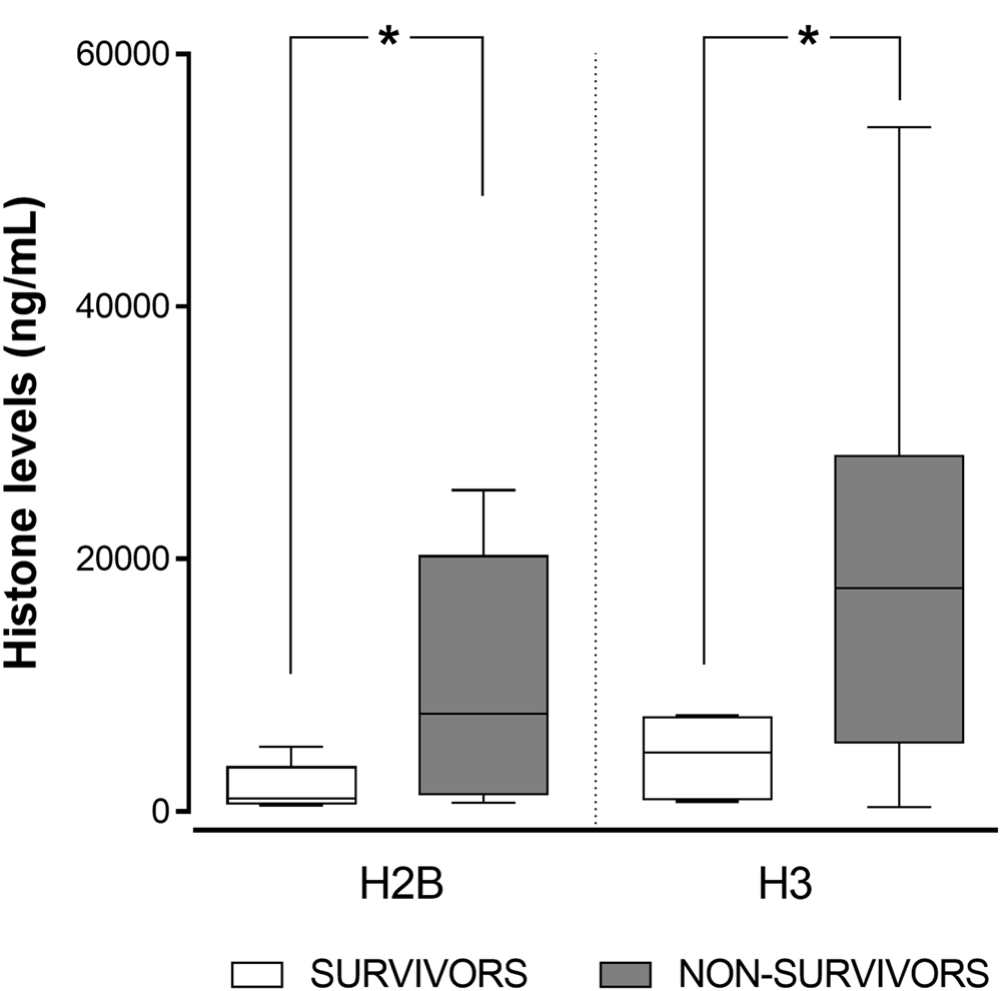
Levels of circulating histones H2B and H3 in the plasma of survivor and non-survivor septic shock patients. Box and whisker plots represent the H2B and H3 levels in the survivors (n = 9) *versus* the non-surviving septic shock patients with bacteraemia (n = 8). Boxes denote interquartile ranges, horizontal lines denote medians, and whiskers denote the 10^th^ and 90^th^ percentiles. Levels are expressed as ng/mL of H2B or H3 in plasma. Differences between both groups were compared using a Student’s *t*-test (*p < 0.05).

We then compared the capacity of circulating histone levels to differentiate the distinct patient subgroups in reference to their final outcome. For the surviving patients (n = 9), the mean level of both histones within the first 24 h in an ICU was significantly lower *versus* the non-survivors (n = 8) (Fig. 3), with up to 10-fold lower values. The mean histone H2B level was 1,351.65 ng/mL (95%CI: 688.01–2,015.28) in the survivors, and 10,106.89 ng/mL (95%CI: 2,076.16–18,137.62) in the non-survivors (p = 0.043). Similar differences were observed for the histone H3 levels, whose mean values were 2,094.31 ng/mL (95%CI: 660.44–3,528.17) for the survivors and 19,988.56 ng/mL (95%CI: 5,743.14–34,233.97) for the non-survivors (p = 0.035) (Fig. 3).

### Specificity and sensitivity of histones H2B and H3 detected by MRM-MS

A ROC curve analysis was performed to evaluate the diagnostic power of the levels of histones H2B and H3 as relevant biomarkers to distinguish septic shock bacteraemia cases from healthy controls. The AUCs (areas under ROC curves), standard error, confidence interval (CI), optimal concentration cut-off value, and sensitivity and specificity percentages for each histone are shown in Table 2.

**Table 2:** ROC curves parameters for histones H2B and H3 levels as biomarkers for diagnosis of septic processes.

Histone	AUC	Standard error	95% CI	p value	Concentration (ng/mL) optimal cutoff value	Sensitivity (%)	Specificity (%)
H2B	0.859	0.070	0.721–0.996	0.002	739.53	82.4	70.0
H3	0.982	0.021	0.942–1.000	<0.0001	574.25	94.1	90.0

Although the AUCs for both histones were similar, and the levels of both biomarkers were significantly different between healthy controls and septic shock patients, histone H3 gave higher values for sensitivity and specificity regarding diagnosis. When a concentration of 574.25 ng/mL was taken as the optimal cut-off value for this biomarker, the sensitivity and specificity of the method were 94.1% and 90.0%, respectively.

### Circulating histones H2B and H3 and their potential for early outcome predictions of septic shock patients

**Figure 4:**
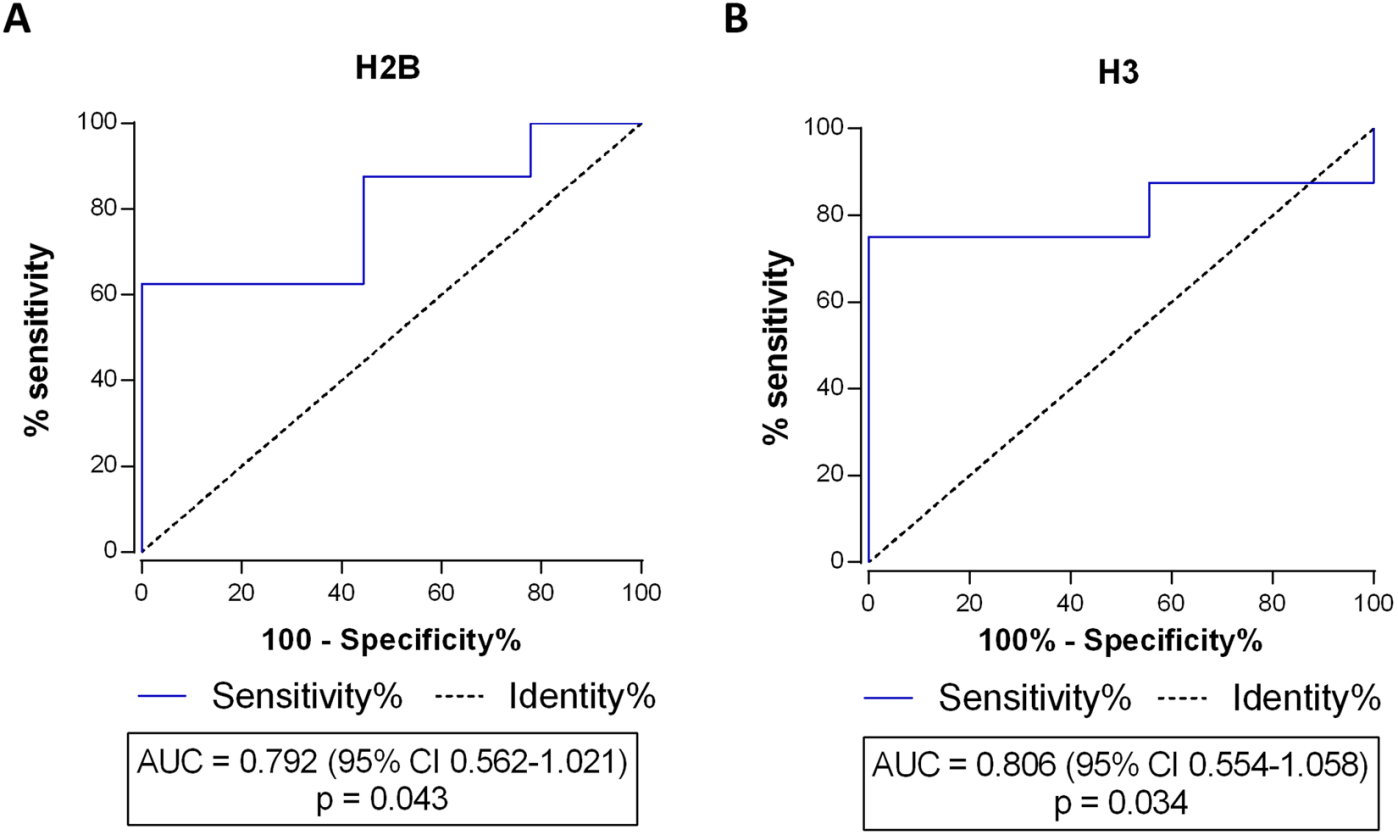
Prognostic potential of circulating histones H2B and H3 for predicting mortality in septic shock patients. Receiver Operating Characteristic (ROC) curves for histone H2B (**A**) and H3 (**B**) based on the MRM-MS quantification of the H2B and H3 levels.

To examine the potential use of histones in plasma as biomarkers for septic shock prognosis, we built the corresponding ROC curve for each histone. Only cases were selected (n = 17), and the optimal cut-off value, sensitivity and specificity to discriminate between the survivors and non-survivors were calculated for both biomarkers (Fig. 4). Once again, histone H3 classified better for prognosis: sensitivity was higher (sensitivity = 75.0%, specificity = 88.9%, cut-off value = 7,559.23 ng/mL) compared to histone H2B (sensitivity = 62.5%, specificity = 88.9%, Cut-off value = 4,784.70 ng/mL).

## Discussion

Early diagnosis and rapid patient stratification of septic shock may improve patient outcome by redefining specific treatment timely and properly. This is a challenge for ICU practitioners, who require new biomarkers to recognise which patients are susceptible to progress towards a more critical pathological condition. The complexities of the sepsis pathophysiology and septic shock, and their many pathological consequences, are the reasons why neither clinical nor biological biomarkers have proven highly accurate to predict adverse outcomes^30^.

MRM-MS is a potential methodology for both biomarker discovery and biomarker validation. This method also provides enough sensitivity and accuracy to validate clinical biomarkers as it does away with the need for further discovery and validation methods^31^. Plasma is a source of biomarkers for diagnostic assays, but is also a challenging biological matrix for proteomic studies because of the wide dynamic range of proteins present in this fluid^32^. Monitoring a single transition per peptide has been proposed as being sufficiently specific for peptide quantitation in complex mixtures such as plasma^33^. Classical biomarkers do not add more information than generic scores to predict outcome in septic patients^34^, and the future treatment of sepsis requires finding new biomarkers that are sensitive, specific and economically affordable.

The overwhelming immune response to infection can release histones into the bloodstream by several mechanisms. Firstly, to fight infection by a mechanism called NETosis. During this process, histones, nucleosomes and other nuclear components are produced by neutrophils in a regulated process that leads to the formation of NETs (Neutrophil Extracellular Traps)^35^. Moreover, histones can be released as a result of endothelial damage^18, 36^ by inducing apoptosis of neutrophils and other immune cells^11^, which contributes to generate a vicious circle.

Toxicity for all histone types has been described, but some related data are controversial. Abrams and collaborators obtained similar cytotoxicity results for both inter-linker histone H1 and core H2A, H2B, H3 and H4 histones^37^, while other authors found higher cytotoxicity for H3, H4, and for H2B in some cases rather than H2A^36, 38^. Furthermore, histones mediate apoptosis of cells in the lymphoid compartment, including the thymus^39^, spleen^40^, and blood^41^. In particular, H4 has been proposed to be the main histone that drives lymphocyte apoptosis. Increased circulating levels of this histone have been related to cellular injury and inflammatory responses observed in human sepsis have also been associated with sepsis-related organ dysfunction^42^. Other studies have identified histone H3 and nucleosomes in plasma from septic patients^36, 43, 44^. Recently, histones have been proposed as the main mediators of cardiac injury in septic shock patients^45^. Cytotoxicity of histones is, however, poorly understood, and data on the molecular details of affected pathways are lacking. In line with this, the TLR4 receptor has been shown to be involved^42^, and we recently described the impairment of vasoactive mediators in endothelial cells as a result of presence of extracellular histones^46^.

Besides histone type, immunoassays have provided different levels of circulating histones in sepsis, but previous studies have never been able to provide a tight range of concentrations that can be considered cytotoxic. In order to identify a sensitive and specific method to quantify circulating histones in plasma samples, we developed an MRM-MS based method that uses Spiked-In peptides LLLPGELAK and STELLIR to detect H2B and H3 histones, respectively.

The present method allows the prediction of the outcome of septic shock patients within the first 24 h of being admitted to an ICU using MS to detect circulating histones as biomarkers for the first time. In this study, the non-survivor patients presented more organ failure (refractory shock, hypoxia, coagulopathy and need for RRT) than the survivors did, as well as significantly higher levels of H3 and H2B in plasma. These findings agree with previously reported results, and provide evidence that plasma histone levels (measured by IA) are associated with sepsis-related organ dysfunction^42^. Hence the present work can contribute to the current clinical approach with more severely and critically ill septic patients by allowing a rapid, novel and accurate measurement of histones at the bedside with prognostic implications.

Despite having developed a novel method, some limitations should be considered, mainly regarding clinical validation. First of all, although the cohort’s sample size may be small for prognostic implications, it can be considered a pilot study of a subset of more severe septic patients (septic shock with bacteraemia), but one that differs from previous studies in that it analyses a larger sample size, but in a highly heterogeneous group (septic population)^8^. Secondly, we employed a control group of healthy volunteers, as previously used by other authors^45^, but it would probably be better in terms of acquiring more information for diagnosis purposes to compare the cohort with critically ill non-infected patients, as in a previous study by Ekaney *et al*.^42^. Lastly, we think that histone content evolution values in a short lapse follow-up could provide us with more prognostic information, but the optimal time frame must be firstly elucidated.

In conclusion, presence of both histones H3 and H2B in plasma can be used for clinical purposes by establishing a specific concentration range by a new spectrometry mass method to be used as a first triage criterion when patients arrive at an ICU. Moreover, they would become valuable prognostic biomarkers of septic shock progression towards a fatal outcome.

## Methods

### Selecting patients and controls

We selected 10 healthy subjects as the control group, and applied the same exclusion criteria as the patients (see below). The control plasma samples were obtained from INCLIVA’s Biobank. For the case group, we included the first 17 consecutive patients with a clinical diagnosis of septic shock upon admission to a medical ICU of the Clinical University Hospital of Valencia (HCUV) (east Spain), and subsequently confirmed bacteraemia (microbiological blood positive culture at 48 h). The exclusion criteria were: i) patients with a life expectancy under 24 h; ii) patients beyond the age range of 18–85 years; iii) patients with an active neoplastic process or treated with antioxidants; iv) patients with a stay in hospital ward prior to ICU admission longer than 24 h or were transferred from another hospital; v) surgical patients. Those patients in a post-cardiopulmonary resuscitation state, or with suspected viral infection, as well as pregnant women and patients who did not provide consent, were excluded. Both patients and controls were enrolled. Informed consent was obtained from all the participants. All the experimental protocols and methods were carried out after obtaining approval from the Biomedical Research Ethics Committee (CEIC) of HCUV. All methods were performed in accordance with the relevant guidelines and regulations.

### Blood collection

Peripheral blood samples were collected using EDTA tubes in both healthy controls and patients. In the latter, blood samples were collected within the first 6 h after being admitted to an ICU. Each sample was centrifuged at 2,500 rpm for 10 min at room temperature (RT) to separate plasma. Then aliquots were stored at −80 °C until used in the MRM-MS experiments.

### Sample preparation

Protein content was measured by the Bradford method (Bio-Rad, Hercules, CA, USA). Firstly, 1 µL of plasma was diluted in 20 µL of a 1:1 ammonium bicarbonate 0.1/trifluoroethanol solution. For each 1 µg of protein content, 300 fmols of both heavy-isotope labelled peptides were added (SpikeTides^TM^ TQL: STELLIR for H3; LLLPGELAK for H2B were prepared as heavy-isotopically labelled proteotypic peptides that terminate with a C-terminal heavy Arg: ^13^C6,^15^N4; Lys: ^1^ ^3^C6,^15^N2) (JPT Peptide Technologies, Berlin, Germany).

Then cysteine residues were reduced by adding 10 mM of D-L‐dithiothreitol at 56 °C for 30 min. The sulfhydryl groups were alkylated with 14 mM of iodoacetamide in the dark at RT for 30 min. The excess was neutralised with 10 mM of D-L‐dithiothreitol in 50 mM of ammonium bicarbonate at a final volume of 100 µL for 30 min at RT. Samples were subjected to trypsin digestion with 1 μg of sequencing grade-modified trypsin (TCPK Trypsin-ABSciex) overnight at 37 °C. The reaction was stopped with trifluoroacetic acid (TFA) at a final concentration of 1%. Samples were dried using speedvac and were resuspended in 50 µL of 0.1% TFA. Peptides were concentrated and purified using ZipTip C18 Tips (Merck Millipore, Darmstadt, Germany). Finally, samples were diluted in 2% acetonitrile (ACN) and 0.1% formic acid (FA) for injections.

### Linearity assessment for Spiked-in peptides

First of all, commercial purified human histones H3 and H2B (EpiGex, Illkirch, France) were trypsinised and analysed using LC-MS/MS_DDA in a 5600 triple TOF (ABSciex, Framingham, MA, USA) to identify those unequivocal peptides with a good signal. From the different fragmentation spectra (MSMS), peptide precursors and fragment ion masses were selected for H3 and H2B to be analysed by MRM-MS. The MRM parameters were optimised in a 5500 QTRAP instrument (ABSciex) by the MRM-PILOT software (AB Sciex) to determine the declustering potential (DP) for each peptide and dwell time (DT) and collision energy for each transition (CE). Afterwards, histone-derived peptides with a large number of detectable transitions, high sensitivity and a wide linear dynamic range were chosen to perform the MRM-MS experiments. The selected peptide sequences (STELLIR for H3; LLLPGELAK for H2B) were used for absolute quantification purposes.

### MRM-MS

The MRM experiments were performed in a 5500 QTRAP hybrid triple quadrupole/linear ion trap mass spectrometer (ABSciex), equipped with an Eksigent 1D + plus NanoLC chromatographic system. Tryptic digest (1 µg of protein and 300 fmol of each Spike-In peptide) was injected into a NanoLC trap column, 3 µ C18-CL (Eksigent, Dublin, CA, USA), and was then separated by RP-HPLC in an analytic NanoLC column 3 µ C18-CL, 15 cm (Eksigent). Chromatography was performed with solvent A (0.1% FA) and solvent B (100% ACN, 0.1% FA) as the mobile phase using a linear gradient (70 min from 2% B to 50% B) at a 300 nL/min flow rate. MRM data were acquired in the positive mode with a spray voltage of 2800 V, curtain gas: 20 psi, ion source gas: 20 psi, interface heater temperature: (IHT) 150 °C, DP: 80, entrance potential (EP): 10, exit potential: (EXP) 15, and a pause time of 3 ms. Collision energy (CE) was 26 V and 23 V for LLLPGELAK - LLLPGELA[K] and STELLIR – STELLI[R], respectively. Transitions (9 for STELLIR and STELLI[R]; and 12 for LLLPGELAK and LLLPGELA[K]) were monitored using Unit Resolution in both quadrupoles Q1 and Q3, and 40 ms of dwell time (DT) for each one.

A data analysis was done and the area ratios (light/heavy) for all the transitions were calculated using the Analyst^®^ 1.5.2 and MultiQuant ^®^ 2.0.2 software (ABsciex).

The H2B and H3 histone levels were estimated based on the average ratio obtained for peptides LLLPGELAK and STELLIR. The final concentrations of H2B and H3 in plasma were calculated from the average signal peak sample/signal peak Spike-In ratio using standard curves constructed by the use of plasma samples from a control individual as complex matrix, increasing concentrations of recombinant human histone H2B and H3, and Spike-In peptides (300 fmol) described above. Standard curves were prepared representing the light-to-heavy (L/H) ratio versus concentration of histones H2B and H3 (fmol). The amount of protein in fmol for each plasma sample was obtained by interpolating L/H ratio for each sample analysed in the standard curve to obtain the concentration of each circulating histone in fmol (see supplementary information). Afterwards, the concentrations of H3 and H2B were obtained by referring the result to the total protein concentration in plasma and the molecular mass (Mr) of the targeted proteins (Mr 13,906 Da and 15,404 Da for H2B and H3, respectively).

### Statistical analysis

The socio-demographic and clinical baseline characteristics of the participants were expressed as continuous variables, with normal distribution expressed as the medians and ranges, means ± standard deviations and categorical data, presented as counts and proportions. For the univariate analysis, differences in clinical data were analysed by the Student’s *t* and chi-square tests. The concentration of histones H2B and H3 were compared by a Student’s *t*-test. The correlation between the H2B and H3 levels was assessed using Pearson’s correlation.

The threshold levels of histones H2B and H3 to be used as biomarkers for the diagnosis and prognosis of septic shock were calculated by a Receiver Operating Characteristic (ROC) curves analysis.

*p* values of < 0.05 were regarded as being statistically significant. All the analyses were conducted by SPSS, v. 20 (IBM Corporation, Armonk, NY, USA).

## Electronic supplementary material

Supplementary information

## References

[1] Seymour CW (2016). Assessment of Clinical Criteria for Sepsis: For the Third International Consensus Definitions for Sepsis and Septic Shock (Sepsis-3). JAMA.

[2] Rhodes A (2017). Surviving Sepsis Campaign: International Guidelines for Management of Sepsis and Septic Shock: 2016. Intensive care medicine.

[3] Torio, C. M. & Andrews, R. M. In *Healthcare Cost and Utilization Project (HCUP) Statistical Briefs* (2006). 21413206

[4] Gaieski DF (2010). Impact of time to antibiotics on survival in patients with severe sepsis or septic shock in whom early goal-directed therapy was initiated in the emergency department. Critical care medicine.

[5] Angus DC, van der Poll T (2013). Severe sepsis and septic shock. The New England journal of medicine.

[6] Pierrakos C, Vincent JL (2010). Sepsis biomarkers: a review. Critical care.

[7] Allam R, Kumar SV, Darisipudi MN, Anders HJ (2014). Extracellular histones in tissue injury and inflammation. J Mol Med (Berl).

[8] Wildhagen KC (2015). Extracellular histone H3 levels are inversely correlated with antithrombin levels and platelet counts and are associated with mortality in sepsis patients. Thromb Res.

[9] Kutcher ME (2012). Extracellular histone release in response to traumatic injury: implications for a compensatory role of activated protein C. J Trauma Acute Care Surg.

[10] Zeerleder S (2012). Circulating nucleosomes and severity of illness in children suffering from meningococcal sepsis treated with protein C. Critical care medicine.

[11] Chen R, Kang R, Fan XG, Tang D (2014). Release and activity of histone in diseases. Cell Death Dis.

[12] Kleine TJ, Lewis PN, Lewis SA (1997). Histone-induced damage of a mammalian epithelium: the role of protein and membrane structure. Am J Physiol.

[13] Hirsch JG (1958). Bactericidal action of histone. J Exp Med.

[14] Gilthorpe JD (2013). Extracellular histone H1 is neurotoxic and drives a pro-inflammatory response in microglia. F1000Res.

[15] Raffray L (2015). Septic shock sera containing circulating histones induce dendritic cell-regulated necrosis in fatal septic shock patients. Critical care medicine.

[16] Romac J, Bouley JP, Van Regenmortel MH (1981). Enzyme-linked immunosorbent assay in the study of histone antigens and nucleosome structure. Anal Biochem.

[17] Costa O, Monier JC (1983). Detection of antibodies to histones in human systemic lupus erythematosus and in murine lupus-like syndromes using micro-ELISA. Ann Immunol (Paris).

[18] José Luis García Giménez, C. R. M., Marta Seco Cervera, J. S. I. C. & Pallardó, F. V. in *Epigenetic* Biomarkers *and Diagnostics* (ed J.L. García-Giménez) Ch. 25, 498-513 (Mica Haley, 2015).

[19] Hoofnagle AN, Wener MH (2009). The fundamental flaws of immunoassays and potential solutions using tandem mass spectrometry. J Immunol Methods.

[20] Rotmensch S, Cole LA (2000). False diagnosis and needless therapy of presumed malignant disease in women with false-positive human chorionic gonadotropin concentrations. Lancet.

[21] Morgan BR, Tarter TH (2001). Serum heterophile antibodies interfere with prostate specific antigen test and result in over treatment in a patient with prostate cancer. J Urol.

[22] Becker JO, Hoofnagle AN (2012). Replacing immunoassays with tryptic digestion-peptide immunoaffinity enrichment and LC-MS/MS. Bioanalysis.

[23] Picotti P (2010). High-throughput generation of selected reaction-monitoring assays for proteins and proteomes. Nat Methods.

[24] Kusebauch U (2016). Human SRMAtlas: A Resource of Targeted Assays to Quantify the Complete Human Proteome. Cell.

[25] Keshishian H, Addona T, Burgess M, Kuhn E, Carr SA (2007). Quantitative, multiplexed assays for low abundance proteins in plasma by targeted mass spectrometry and stable isotope dilution. Mol Cell Proteomics.

[26] Bronsema KJ, Bischoff R, van de Merbel NC (2013). High-sensitivity LC-MS/MS quantification of peptides and proteins in complex biological samples: the impact of enzymatic digestion and internal standard selection on method performance. Analytical chemistry.

[27] Gao J (2014). Absolute quantification of histone PTM marks by MRM-based LC-MS/MS. Analytical chemistry.

[28] Anderson KW, Mast N, Pikuleva IA, Turko IV (2015). Histone H3 Ser57 and Thr58 phosphorylation in the brain of 5XFAD mice. FEBS open bio.

[29] Matsuda S, Furuya K, Ikura M, Matsuda T, Ikura T (2015). Absolute quantification of acetylation and phosphorylation of the histone variant H2AX upon ionizing radiation reveals distinct cellular responses in two cancer cell lines. Radiation and environmental biophysics.

[30] Garcia-Simon M (2015). Prognosis Biomarkers of Severe Sepsis and Septic Shock by 1H NMR Urine Metabolomics in the Intensive Care Unit. PLoS One.

[31] Cohen Freue GV, Borchers CH (2012). Multiple reaction monitoring (MRM): principles and application to coronary artery disease. Circ Cardiovasc Genet.

[32] Anderson NL, Anderson NG (2002). The human plasma proteome: history, character, and diagnostic prospects. Mol Cell Proteomics.

[33] Gerber SA, Rush J, Stemman O, Kirschner MW, Gygi SP (2003). Absolute quantification of proteins and phosphoproteins from cell lysates by tandem MS. Proc Natl Acad Sci USA.

[34] Antonelli M (2009). Year in review in Intensive Care Medicine, 2008: III. Paediatrics, ethics, outcome research and critical care organization, sedation, pharmacology and miscellanea. Intensive care medicine.

[35] Brinkmann V (2004). Neutrophil extracellular traps kill bacteria. Science.

[36] Xu J (2009). Extracellular histones are major mediators of death in sepsis. Nat Med.

[37] Abrams ST (2013). Human CRP defends against the toxicity of circulating histones. J Immunol.

[38] Mena HA (2016). Extracellular histones reduce survival and angiogenic responses of late outgrowth progenitor and mature endothelial cells. J Thromb Haemost.

[39] Wang SD, Huang KJ, Lin YS, Lei HY (1994). Sepsis-induced apoptosis of the thymocytes in mice. J Immunol.

[40] Ayala A (1999). Increased inducible apoptosis in CD4 + T lymphocytes during polymicrobial sepsis is mediated by Fas ligand and not endotoxin. Immunology.

[41] Le Tulzo Y (2002). Early circulating lymphocyte apoptosis in human septic shock is associated with poor outcome. Shock.

[42] Ekaney ML (2014). Impact of plasma histones in human sepsis and their contribution to cellular injury and inflammation. Critical care.

[43] Xu J, Ji Y, Zhang X, Drake M, Esmon CT (2009). Endogenous activated protein C signaling is critical to protection of mice from lipopolysaccaride-induced septic shock. J Thromb Haemost.

[44] Abrams ST (2013). Circulating histones are mediators of trauma-associated lung injury. Am J Respir Crit Care Med.

[45] Alhamdi Y (2015). Circulating Histones Are Major Mediators of Cardiac Injury in Patients With Sepsis. Critical care medicine.

[46] Pérez-Cremades, D. B.-B. *et al*. Extracellular histones disarrange vasoactive mediators release through a COX-NOS interaction in human endothelial cells. *Journal of cellular and molecular medicine* (in press), doi:10.1111/jcmm.13088 (2016). 10.1111/jcmm.13088 PMC5543457 28244682

