# Supplementary material for: A new mass spectrometry-based method for the quantification of histones in plasma from septic shock patients
Source: Sci Rep. 2017 Sep 6;7:10643. doi: 10.1038/s41598-017-10830-z (PMC5587716; doi:10.1038/s41598-017-10830-z)
Supplement: Supplementary file 1 — Supplementary information [file 41598_2017_10830_MOESM1_ESM.pdf]

A new mass spectrometry-based method for the quantification of histones in plasma from septic shock patients

García-Giménez J.L. [PhD, 1,2,3,4*], Romá-Mateo C.[PhD, 1,2,3,4,10], Carbonell N.[MD, PhD, 3,5], Palacios L.[MD, 3,5], Peiró-Chova L.[PhD, 3, 6], García-López E.[PhD, 2], García-Simón M.[MD,PhD 3,5], Lahuerta R.[MD, 3,5], Gimenez-Garzó C.[PhD, 2,3], Berenguer-Pascual E.[2,4],Mora M.I. [7], Valero M.L.,[8] Alpízar A.,[9] Corrales F.J.,[9] Blanquer J.[MD, PhD, 3,5], Pallardó F.V.[MD, PhD, 1,2,3,4*].

[1] Center for Biomedical Network Research on Rare Diseases (CIBERER). Institute of Health Carlos III. Valencia (Spain)

[2] Department of Physiology. Faculty of Medicine and Dentistry. University of Valencia. Valencia (Spain).

[3] INCLIVA Biomedical Research Institute. Valencia (Spain)

[4] Epigenetics Research Platform. CIBERER/UV. Valencia (Spain)

[5] Intensive Care Unit, Clinical University Hospital of Valencia. Valencia (Spain)

[6] INCLIVA Biobank. INCLIVA Biomedical Research Institute. Valencia (Spain)

[7] Department of Hepatology, Proteomics laboratory. CIMA, University of Navarra (Spain); Ciberhed; Idisna; PRB2, ProteoRed-ISCIII

[8] Central Service for Experimental Research (SCSIE). University of Valencia. Burjassot (Spain).

[9] Proteomics Unit . Centro Nacional de Biotecnología (CSIC); PRB2, ProteoRed-ISCIII

[10]Faculty of Biomedical and Health Sciences. Universidad Europea de Valencia. Valencia (Spain)

Corresponding authors

Dr. José Luis García Giménez and Prof. Federico V. Pallardó

Department of Physiology. Faculty of Medicine and Dentistry. University of Valencia. Valencia (Spain). CIBERER. Biomedical Network Research Center for Rare Diseases. INCLIVA Biomedical Research Institute. Avda. Blasco Ibañez Nº 15. 46010 - Valencia, Spain. Phone:+34963864646

E-mail: j.luis.garcia@uv.es
federico.v.pallardo@uv.es

Supplementary information

A standard curve was prepared with plasma samples from control subjects containing increasing concentrations of commercial H2b and H3 (EpiGex, Illkirch, France) and a fixed concentration (300 fmol) of SpikeTides^{TM} TQL: STELLIR for H3; LLLPGELAK for H2B peptides (JPT Peptide Technologies, Berlin, Germany). Samples were then digested with trypsin and analysed following the procedures described for the target samples in Methods section. Standard curves were prepared representing the light-to-heavy (L/H) ratio versus concentration of histones H2B and H3 (fmol). Linear regression equation for each histone deduced from the mathematical fitting of the histone standard calibration data were used for calculating endogenous circulating H2B and H3 amounts (fmol) in each sample as described in Methods section.

[Histone H2B recombinant]	Ratio L/H
3,125	0,00579021
6,25	0,00892438
12,5	0,01359195
15,625	0,01534174
25	0,02492153
31,25	0,02968093
62,5	0,05874242
125	0,12094489
250	0,25289138
500	0,49999022

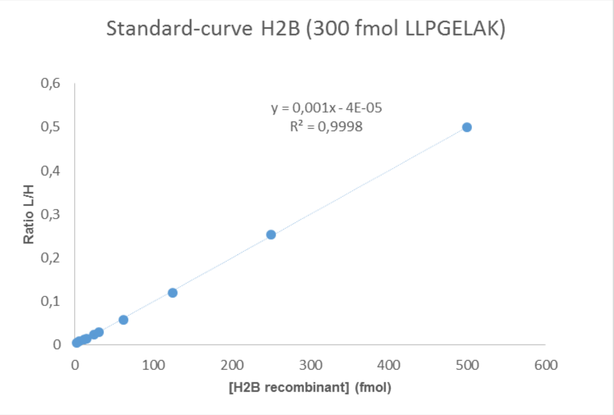

Supplementary Figure S1. Standard curve calculated from the mathematical fitting of the recombinant histone H2B standard concentration and L/H ratio calculated by MRM-MS. L/H ratio was obtained of integrated peak areas from the signal corresponding to transitions (heavy, 300 fmol and increasing amounts (fmol) of recombinant histone H2B.

[Histone H3 recombinant] fmol	Ratio L/H
3,125	0,002767415
6,25	0,005048136
12,5	0,007939073
15,625	0,009520891
25	0,013837216
31,25	0,018275997
62,5	0,033268333
125	0,066676949
250	0,135985603
500	0,280923357

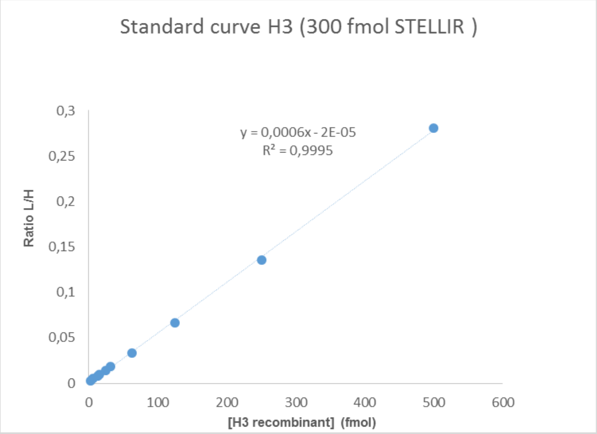

Supplementary Figure S2. Standard curve calculated from the mathematical fitting of the recombinant histone H3 standard concentration and L/H ratio determined by MRM-MS. Chromatograms for peptides STELLIR showing the integrated peak areas from the signal corresponding to transitions (heavy, 300 fmol and increasing amounts (fmol) of recombinant histone H3).
